# Respiratory Burst Oxidase Homologs RBOHD and RBOHF as Key Modulating Components of Response in Turnip Mosaic Virus—*Arabidopsis* *thaliana* (L.) Heyhn System

**DOI:** 10.3390/ijms21228510

**Published:** 2020-11-12

**Authors:** Katarzyna Otulak-Kozieł, Edmund Kozieł, Józef Julian Bujarski, Justyna Frankowska-Łukawska, Miguel Angel Torres

**Affiliations:** 1 Department of Botany, Institute of Biology, Warsaw University of Life Sciences—SGGW, Nowoursynowska Street 159, 02-776 Warsaw, Poland; justyna_frankowska_lukawska@sggw.edu.pl; 2 Department of Biological Sciences, Northern Illinois University, DeKalb, IL 60115, USA; jbujarski@niu.edu; 3 Centro de Biotecnología y Genómica de Plantas (CBGP), Universidad Politécnica de Madrid (UPM)—Instituto Nacional de Investigacióny Tecnología Agraria y Alimentaria (INIA), Campus de Montegancedo, 28223 Pozuelo de Alarcón (Madrid), Spain; miguelangel.torres@upm.es; 4 Departamento deBiotecnología-Biología Vegetal, Escuela Técnica Superior de Ingeniería Agronómica, Alimentaria y de Biosistemas, 28040 Madrid, Spain

**Keywords:** plant–virus interaction, resistance response, potyvirus, ultrastructure, pathogenesis-related protein 1, hydrogen peroxide

## Abstract

Turnip mosaic virus (TuMV) is one of the most important plant viruses worldwide. It has a very wide host range infecting at least 318 species in over 43 families, such as Brassicaceae, Fabaceae, Asteraceae, or Chenopodiaceae from dicotyledons. Plant NADPH oxidases, the respiratory burst oxidase homologues (RBOHs), are a major source of reactive oxygen species (ROS) during plant–microbe interactions. The functions of RBOHs in different plant–pathogen interactions have been analyzed using knockout mutants, but little focus has been given to plant–virus responses. Therefore, in this work we tested the response after mechanical inoculation with TuMV in *Arabidopsis* *rbohD* and *rbohF* transposon knockout mutants and analyzed ultrastructural changes after TuMV inoculation. The development of the TuMV infection cycle was promoted in *rbohD* plants, suggesting that RbohD plays a role in the *Arabidopsis* resistance response to TuMV. *rbohF* and *rbohD/F* mutants display less TuMV accumulation and a lack of virus cytoplasmic inclusions were observed; these observations suggest that RbohF promotes viral replication and increases susceptibility to TuMV. *rbohD/F* displayed a reduction in H_2_O_2_ but enhanced resistance similarly to *rbohF*. This dominant effect of the *rbohF* mutation could indicate that RbohF acts as a susceptibility factor. Induction of hydrogen peroxide by TuMV was partially compromised in *rbohD* mutants whereas it was almost completely abolished in *rbohD/F,* indicating that these oxidases are responsible for most of the ROS produced in this interaction. The pattern of in situ H_2_O_2_ deposition after infection of the more resistant *rbohF* and *rbohD/F* genotypes suggests a putative role of these species on systemic signal transport. The ultrastructural localization and quantification of pathogenesis-related protein 1 (PR1) indicate that ROS produced by these oxidases also influence PR1 distribution in the TuMV-*A.* *thaliana* pathosystem. Our results revealed the highest activation of PR1 in *rbohD* and Col-0. Thus, our findings indicate a correlation between PR1 accumulation and susceptibility to TuMV. The specific localization of PR1 in the most resistant genotypes after TuMV inoculation may indicate a connection of PR1 induction with susceptibility, which may be characteristic for this pathosystem. Our results clearly indicate the importance of NADPH oxidases RbohD and RbohF in the regulation of the TuMV infection cycle in *Arabidopsis*. These findings may help provide a better understanding of the mechanisms modulating *A.* *thaliana*–TuMV interactions.

## 1. Introduction

Turnip mosaic virus (TuMV) belongs to the genus Potyvirus (family *Potyviridae*), a virus with a single (+) RNA of approximately 10 kb encoding a polyprotein, which is processed by three virus endogenous proteinases into 12 proteins [1]. TuMV has an unusually wide host range, which is of particular importance to Brassicaceae plants including Arabidopsis, but this is the most damaging in *Brassica* *rapa* subsp. *pekinensis* and *chinensis* (L.), H. Bailey, *Brassica* *rapa* (L.), *Sinapis* sp. and *Raphanus sativus* (L.). It also attacks *Beta vulgaris* (L.), *Spinacia oleracea* (L.) and *Nicotiana tabacum* (L.) [2]. TuMV’s economic importance varies depending on crop and location. It has a devastating effect on crops in China, where infection with it is widespread [3,4,5]. Examples of quantified yield losses include 30% in *B. napobrassica* crops in Southern Ontario [5,6] and 70% in infected *B. napus* in England and Wales [5,7]. TuMV also causes quality defects such as internal necrotic disorders which account for around 10% of losses in stored *B. oleracea* var. capitata [5].

Potyvirus particles are organized as a flexous rod in length and are between 11 and 15 nm in diameter. Akin to other (+) RNA viruses, at the ultrastructural level TuMV causes remodeling of the host cellular membranes to create viral structures called “viral factories” [8]. In the case of TuMV, Cotton et al. [9] documented that infection leads to the formation of vesicles originating from the ER. Virus protein 6K2 induces the formation of these vesicles containing the viral RNA as well as several replication-related host and virus proteins that as a whole are able to act as potential replication sites [10]. Moreover, Grangeon et al. [11] and Wan et al. [12] also postulated that vesicles are associated with TuMV cell-to-cell as well as long distance movement. Furthermore, Movahed et al. [13] demonstrated the presence of host factors participating in the plant immune response in purified vesicles from *N. benthamiana* and Arabidopsis, together with viral proteins. Secondly, important ultrastructural changes observed during TuMV infection are due to the presence of filamentous viral particles (VPs) and viral cytoplasmatic inclusion (CI) [2]. Generally, TuMV isolates have been frequently characterized using a variety of differential plant lines from various brassica species [2,14]. However, the first resistance gene against TuMV to be mapped, *Tu*, was identified in lettuce cv. Cobbham Green [15]. *TuRB01* was the first TuMV resistance gene to be mapped in brassicas, in the *Brassica napus* line N-o-1 [16]. Among the four *B.napus* lines used by Jenner and Walsh [2,17], line R4 contains a single dominant resistance gene effective against the isolate TuMV UK1 [18] and other pathotype 1 isolates [16]. Coutts et al. [19] identified, in Australian varieties of a population of *Brassica napus* and *Brassica juncea* plants, TuMV resistance genes *TuRB01*, *TuRB03*, *TuRB04* and *TuRB05*. In the case of *Arabidopsis,* resistance is acquired mostly by the generation of different types of mutants. TuMV infection is not supported efficiently after silencing of *HSP70* [20,21]. Knockout mutation in the *DNA-binding protein phosphatase 1* (*DBP1*) gene does not influence plant growth in *Arabidopsis*, but does result in resistance to two potyviruses—TuMV and plum pox virus (PPV) [22]. Despite known resistance genes, TuMV is still serious and a current danger for crop production [21].

The formation of reactive oxygen species (ROS; superoxide anion, hydrogen peroxide, hydroxyl radical and singlet oxygen) is a regular process in the metabolism of aerobic organisms [16]. These ROS were initially thought to be also harmful molecules [17], inducing irreversible cell damage or even cause cell death [23]. To prevent damage, plants and other organisms have developed effective antioxidant systems, and the dynamic balance of ROS is maintained through multiple pathways and approaches [24,25]. However, plants and other organisms harbor enzymes for a regulated generation of superoxide and hydrogen peroxide that have been postulated as signals to react to abiotic and biotic stresses [26]. The generation of ROS during the pathogen response is mainly dependent upon different respiratory bursts of oxidase homologs, which are activated by pathogen recognition, leading to the establishment of the immune response often associated with the hypersensitive response [27]. Similarly to other pathogens, virus infection leads to a rapid production of reactive oxygen species (ROS). A plant–virus interaction was one of the first pathosystems where this so called oxidative burst was discovered—the *Nicotiana*-*Tobacco mosaic virus* pathosystem [28]. Plant NADPH oxidases, known as Respiratory Burst Oxidase Homologs (RBOHs) are responsible for this ROS generation [29]. RBOHs constitute a family of plasma membrane–localized enzymes with homology to the mammalian phagocyte NADPH oxidase, NOX2 [30]. They generate superoxide anions (O_2_^-^) in the apoplast which are rapidly dismutated to hydrogen peroxide (H_2_O_2_). The model plant *Arabidopsis thaliana* presents a multiple family of 10 *Rboh* genes [31]. Analyses by Suzuki et al. [32] demonstrated that *Rboh* genes control a wide range of developmental as well as environmental processes. Two of these genes, *RbohD* and *RbohF,* are pleiotropic and function together, mediating diverse functions in abiotic stress signaling as well in pathogen responses. These two genes present a different expression pattern during plant development and are differentially modulated during the immune response [29]. Moreover, RBOHD and RBOHF proteins are differentially activated by different protein kinases [33,34]. Furthermore, RBOH-derived ROS appear to trigger local and/or systemic reprogramming to activate plant immunity [35].

Even though the roles of *RbohD* and *RbohF* have been extensively documented in many plant–pathogen interactions, the potential function(s) of Rboh-mediated responses during plant–virus interactions remain poorly investigated. The aim of this research was to perform *Turnip mosaic virus* inoculation on *Arabidopsis rbohD* and *rbohF* mutant plants to assess the role of these NADPH oxidases in this pathosystem. We want to examine whether RbohD and RbohF contribute to signal transduction crosstalk in TuMV–*Arabidopsis* interactions.

## 2. Results

### 2.1. Accumulation of TuMV in Wild Type and Mutant Arabidopsis thaliana Plants

**Figure 1:**
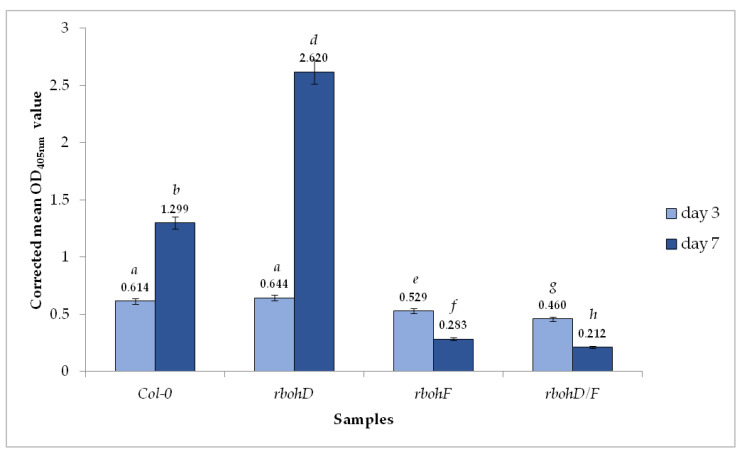
TuMV detection and relative virus concentration assessment in *Arabidopsis thaliana* Col-0 and *rbohD*, *rbohF* and *rbohD/F* mutants at 3 and 7 days after inoculation. Values represented are mean of corrected OD_405nm_ values. Significant differences between classes at *p* < 0.05 level of significance were assessed by analysis of variance (ANOVA) with post-hoc Tukey HSD. The statistically significant values are marked by letters above chart bars.

The double antibody sandwich enzyme-linked immunosorbent assay (DAS-ELISA) clearly showed the presence of TuMV in all *Arabidopsis thaliana* wild type Col-0 and the three *rboh* mutants at 3 and 7 days post-inoculation (dpi). However, OD_405_ values were higher in the Col-0 and *rbohD* plants than in *rbohF* and double *rbohD/F* mutants (Table 1). Moreover, whereas the values for Col-0 and *rbohD* increased between 3 and 7 dpi (to 1.379 and 2.701, respectively), the *rbohF* and double mutant *rbohD/F* OD_405_ values significantly decreased at a later time (Table 1). Analyses using the corrected OD_405nm_ mean values confirmed the increase in relative concentration of TuMV by 2.11-fold in Col-0 plants and over 4.0-fold in *rbohD*, and the decrease in the relative virus amount between 3 and 7 dpi, the decrease in *rbohF* by 1.86-fold and by 2.16-fold in *rbohD/F* (Figure 1). Moreover, the virus concentration at the 3-dpi time point was lower in *rbohF*, and especially in *rbohD/F* than in Col-0 or *rbohD* mutants. These results with a boosted TuMV infection cycle in *rbohD* mutants suggest that this oxidase contributes to stopping virus progression.

### 2.2. RbohD and RbohF in TuMV Infection

**Figure 2:**
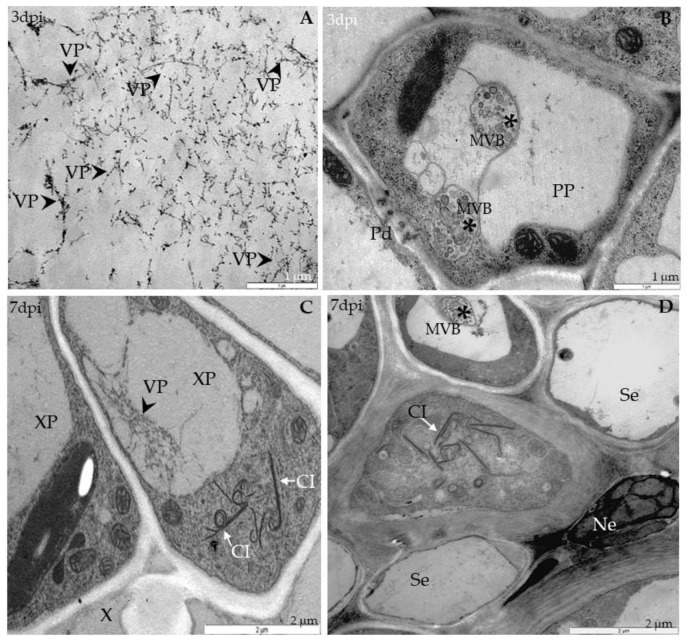
Viral particles and inclusions, multivesicular bodies induction and chloroplast changes in Col-0 different leaf tissues 3 (**A**,**B**) and 7 days (**C**,**D**) after TuMV inoculation examined with a transmission electron microscope (TEM, FEI M268D “Morgagni” transmission electron microscope). (**A**) Virus particles (VPs; marked by arrowhead) in vacuole (v) of palisade mesophyll cell. (**B**) Multivesicular bodies (MVBs; marked by *) in cytoplasm and vacuole (v) of phloem parenchyma (PP) cell. Pd-plasmodesmata. (**C**) Virus particles (VPs; arrowhead) in vacuole (v) and virus inclusions (cytoplasmatic inclusion (CI); marked by arrow) in cytoplasm of xylem parenchyma (XP) cell. (**D**) Virus cytoplasmic inclusions (Cis; arrow) inside phloem cell and necrotic changes (Ne) in companion cell (CC). Se—sieve element; X—xylem tracheary element.

**Figure 3:**
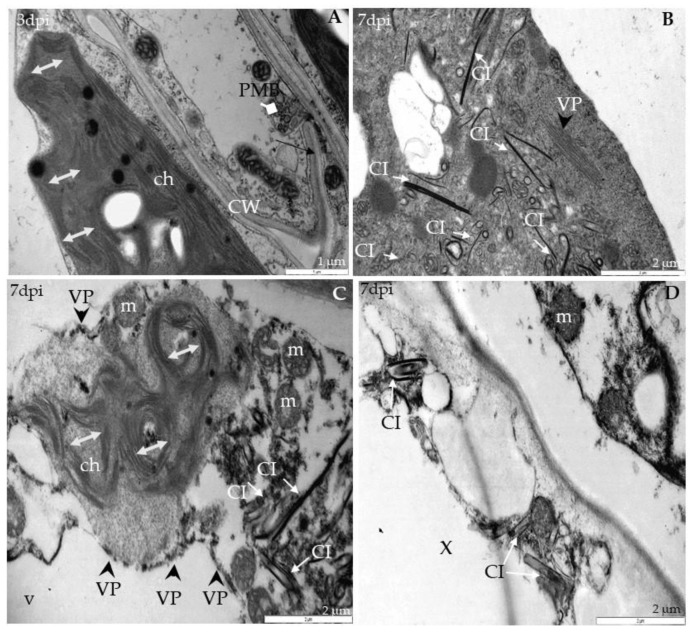
Virus particles and viral cytoplasmatic inclusion, changes in chloroplast lamellae stacks in *rbohD* plants leaf tissues 3 (**A**) and 7 days **(B**–**D**) after TuMV inoculation examined under TEM. (**A**) Curved (double-headed arrow) chloroplast (ch) thylakoids, cell wall (CW) invagination (black arrow) and paramular bodies (PMBs; marked by diamond head arrow) in palisade mesophyll cell. (**B**) Virus particles (VPs, with arrowhead) and virus cytoplasmic inclusions (CIs; arrows) in palisade mesophyll cell. (**C**) Destroyed chloroplast (ch) with disorganized thylakoids (double-headed arrow), virus cytoplasmic inclusions (CIs; white arrow) and viral particles (VPs; black arrowhead) in cytoplasm of spongy mesophyll cell. v—vacuole; m—mitochondrion. (**D**) Virus inclusions (CI; arrow) inside xylem tracheary elements (X). m—mitochondrion.

Ultrastructural analyses of Col-0, *rbohD*, *rbohF* and double mutant *rbohD/F* at 0 to 7 days after TuMV inoculation, as well as in mock-inoculated plants, were examined. In Col-0, inoculated plants’ virus particles were first noticed starting from 3 dpi, and were mainly located inside the vacuoles of mesophyll cells (Figure 2A). This accumulation was accompanied by the appearance of multivesicular structures in mesophyll and phloem tissues (Figure 2B). In vascular tissues of inoculated rosette leaves, a few virus cytoplasmic inclusions in xylem parenchyma (Figure 2C) as well as in phloem cells were observed (Figure 2D). Phloem cells very often revealed necrotization at 7 days after inoculation, whereas in xylem parenchyma cells virus particles in vacuoles were presented. The analysis of *rbohD* mutants confirmed important disorders in chloroplast structures. Curved structures of thylakoids in chloroplasts were observed compared with mock-inoculated leaves (Figure 3A); at 7 dpi the alterations were more intense than in Col-0.

These changes were accompanied by cell wall invagination as well as paramular body induction, especially in palisade mesophyll cells (Figure 3A). Moreover, virus particles and virus-induced cytoplasmic inclusions were the most frequently presented in *rbohD* mesophyll as well as in phloem parenchyma cells 7 days after virus inoculation (Figure 3B,C). Furthermore, in *rbohD* mesophyll cells virus particles were attached to the tonoplast, which was usually accompanied by disorganized chloroplast structures (with curved thylakoids stacks) and virus cytoplasmic inclusion present in cytoplasm (Figure 3C). Chloroplast changes in *rbohD* were definitely more intense than in Col-0 leaves’ mesophyll cells. Moreover, contrary to the Col-0 xylem cells, virus inclusions occurred commonly inside xylem tracheary elements (Figure 3D). These observations indicated that *rbohD* displays more viral particles and ultrastructural alterations in response to TuMV inoculation than Col-0.

**Figure 4:**
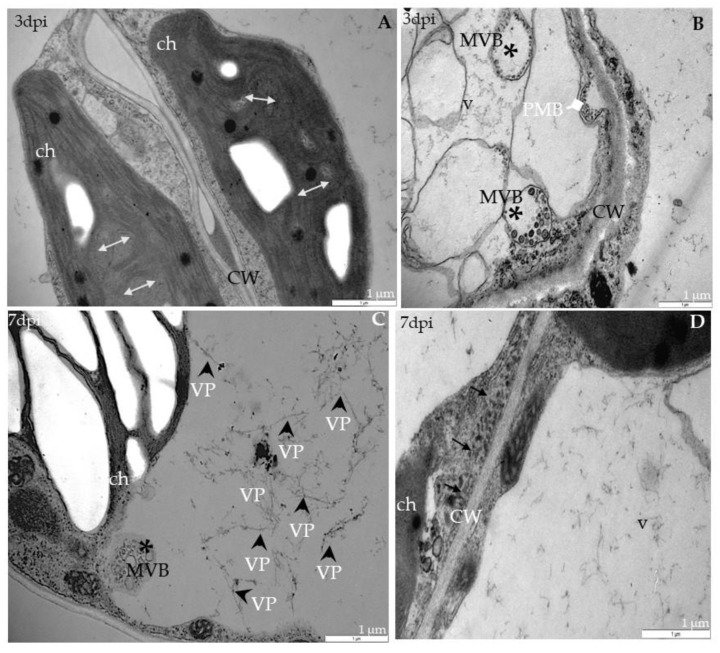
Curved structure of chloroplast lamellae stacks—multivesicular structures and viral particles in vacuole in different leaf tissues of *rbohF* plants 3 (**A**,**B**) and 7 days (**C**,**D**) after TuMV inoculation examined under a TEM. (**A**) Curved thylakoids (double-headed arrow) in chloroplast (ch) of palisade mesophyll cell. (**B**) Multivesicular structures (“single membranes with tubules”) (MVB; *) in vacuole (v) and paramular bodies (PMBs; marked by diamond head arrow) in phloem apoplast area. CW—cell wall. (**C**) Virus particles (VPs; arrowhead) and multivesicular bodies (MVB; *) inside vacuole (v) of spongy mesophyll cell. ch—chloroplast. (**D**) Irregular structure (arrows) of the cell wall (CW) between spongy mesophyll cells. ch—chloroplast; v—vacuole.

**Figure 5:**
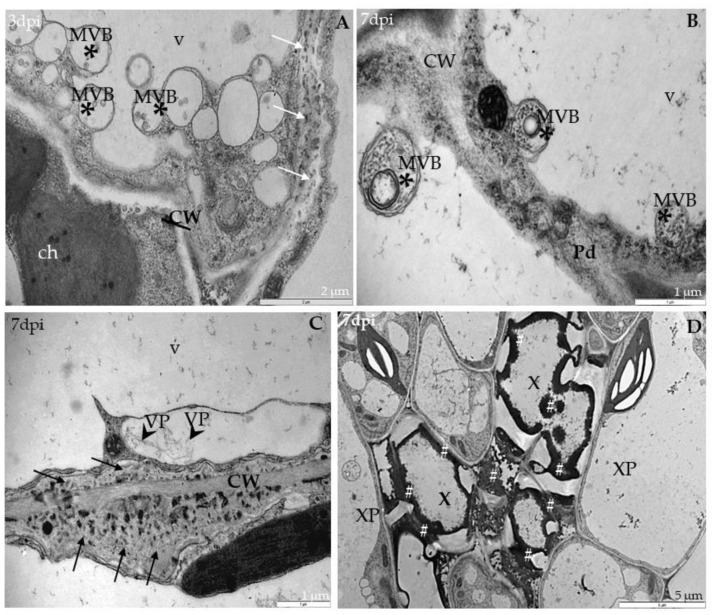
Multivesicular bodies’ induction, viral particles in vacuole and cell wall rebuilding in *rbohD/F* plants tissues 3 (**A**) and 7 days (**B**–**D**) after TuMV inoculation examined under a TEM. (**A**) Cell wall rebuilding (CW; arrows) and multivesicular bodies (MVB; *) in phloem parenchyma cells. Ch—chloroplast; V—vacuole. (**B**) Multivesicular bodies (MVBs; *) near plasmodesmata (Pd) in phloem parenchyma cells. CW—cell wall; V—vacuole. (**C**) Cell wall (CW) rebuilding (arrows) and virus particles (VPs; arrowhead) in membranous structures such as small vacuoles in palisade mesophyll cells. V—vacuole. (**D**) Accumulation of phenolic-like compounds (marked with #) inside xylem tracheary elements (X). XP—xylem parenchyma.

In *rbohF*-TuMV-inoculated plants, starting from 3 dpi, a slight curve of chloroplast thylakoids was noticed compared with mock-inoculated plants (Figure 4A). Moreover, membranous structures and single membranes with tubules or paramular bodies in the apoplast were induced 3 days after TuMV inoculation (Figure 4B). In *rbohF*, a lower presence of virus particles’ compared with *rbohD* or even with Col-0 tissues was noticed (Figure 4C), whereas cell wall rebuilding (such as reinforcing) was clearly more visible at 7dpi than in Col-0 and *rbohD* tissues (Figure 4D). On the contrary, in rbohD/F, a double mutant structural alteration in the cell wall compared with mock-*rbohD/F* was already presented starting at 3 dpi (Figure 5A). At day 7 in this mutant, the multivesicular bodies were induced in phloem parenchyma cells (Figure 5A,B). Moreover, only a few virus particles were noticed, with a lower frequency, inside small vacuoles in mesophyll cells (Figure 5C). As for the cell wall structure, it was more intensively rebuilt (reinforced) at 7 dpi (Figure 5C) than at 3 dpi as well as than it was in *rbohF* tissues. Furthermore, along cell walls, phenolic-like compounds were detected in *rbohD/F* at the 7-dpi time point (Figure 5D). Neither virus particles nor virus cytoplasmic inclusions were observed in *rbohD/F*’s phloem (Figure 5A,B) and xylem cells (Figure 5D).

These observations indicated that *rbohF* displays fewer virus particles than *rbohD* tissues and Col-0. Although double mutant *rbohD/F* revealed the same phenotype as *rbohF*, it seems *rbohF* supports fewer TuMV proliferations. Therefore, taking into account the relative virus concentration as well as ultrastructural analyses, it can be summarized that RbohD is required to limit virus proliferation, whereas RbohF must have a different role allowing virus proliferation and acts dominantly toward RbohD.

### 2.3. Localization of TuMV

**Figure 6:**
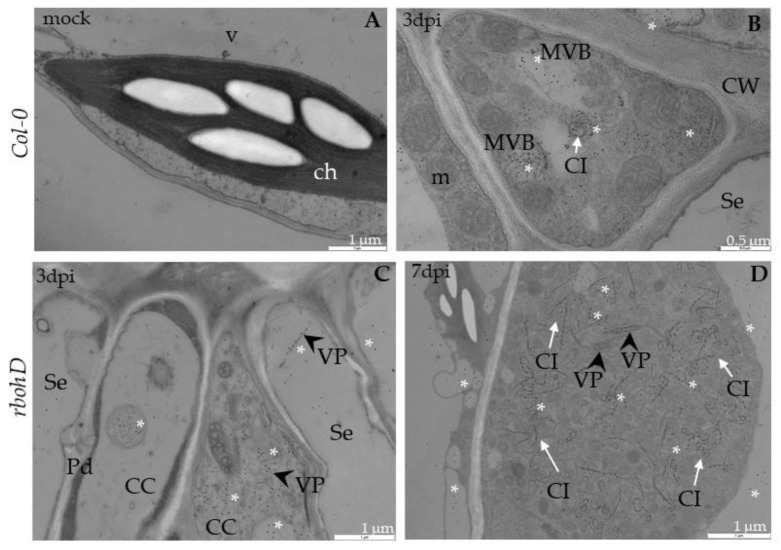
TuMV detection in Col-0 and *rbohD* mock-inoculated leaf tissues (**A**) and 3 (**B**,**C**) and 7 days (**D**) after virus inoculation by immunogold electron microscopy. (**A**) Unchanged palisade mesophyll cell without TuMV epitope from mock-inoculated Col-0 plants. ch—chloroplast; v—vacuole. (**B**) TuMV epitope presence (*) around cytoplasmic inclusion (CI; arrow) and multivesicular bodies (MVBs) in phloem parenchyma cell of Col-0. CW—cell wall; m—mitochondrion; Se—sieve tube. (**C**) TuMV epitope localization (*) along virus particles (VPs; arrowhead) in companion cell (CC) and sieve element (Se) in *rbohD* phloem tissue. Pd—plasmodesmata. (**D**) TuMV epitope presence (*) around virus particles (VPs; arrowhead) and cytoplasmic inclusion (CI; arrow) in vesicular structures in *rbohD* palisade mesophyll cells.

**Figure 7:**
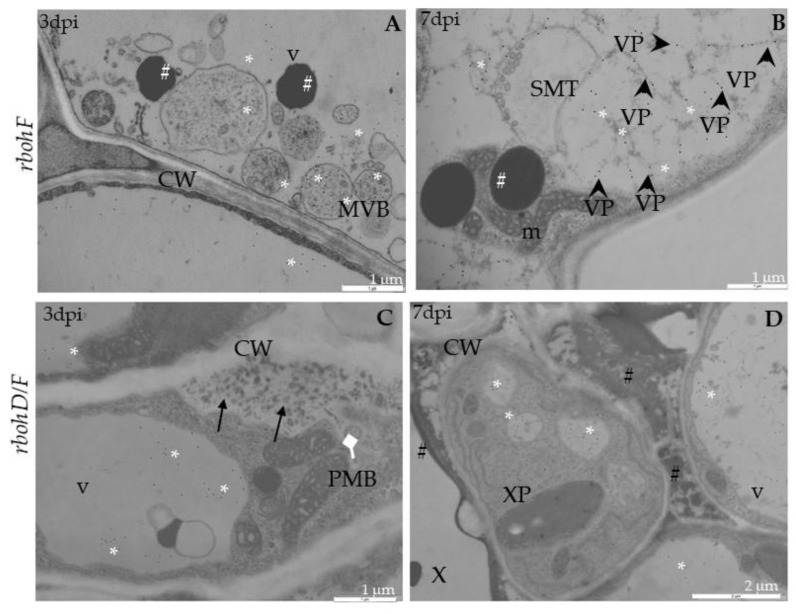
TuMV detection in *rbohF* and *rbohD/F* leaf tissues 3 (**A**,**C**) and 7 days (**B**,**D**) after virus inoculation examined by immunogold electron microscopy. (**A**) TuMV epitope localization (*) around multivesicular structures (MVB) in *rbohF* phloem cells. Phenolic-like compounds (#) near MVB. CW—cell wall; v—vacuole. (**B**) TuMV epitope presence (*) along virus particles (VPs; arrowheads) in vacuole (v) and around single membrane with tubule structures (SMTs) in *rbohF*. Phenolic-like compounds (#). m—mitochondrion. (**C**) TuMV epitope (*) in vacuole (V) and around changed (black arrows) cell wall (CW) with paramular bodies (PMBs; diamond head arrow) in *rbohD/F* phloem parenchyma cell. (**D**) TuMV epitope (*) in vacuole (v) *rbohD/F* xylem parenchyma cells (XP). Phenolic-like compounds (#) inside xylem tracheary elements (X).

**Figure 8:**
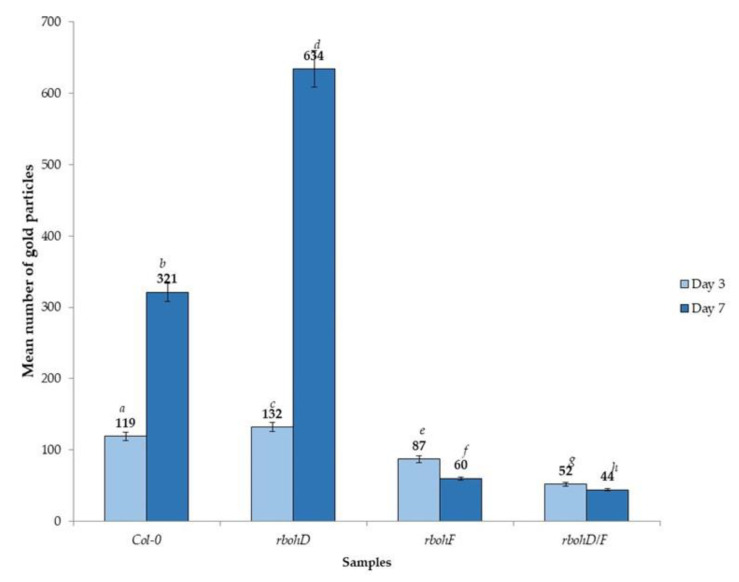
Quantification of gold particles associated with TuMV in *Arabidopsis thaliana* Col-0, *rbohD*, *rbohF* and double *rbohD/F* mutants at 3 and 7 days after inoculation. Significant differences between classes at *p* < 0.05 level of significance by ANOVA with post-hoc Tukey HSD were assessed. The statistical significant values are marked by letters above chart bars.

Besides analyzing the relative virus concentration, we monitored the TuMV localization in situ in Col-0 and *rboh* mutants at the ultrastructural level (Figure 6, Figure 7 and Figure 8). There were no gold depositions noticed neither in control mock-inoculated tissues (Figure 6A) nor in sections where primary antibodies were replaced with the pre-immune serum. In Col-0, the epitopes were noticed in the cytoplasm near the inclusions (Figure 6B). Virus epitopes were co-localized in the cytoplasmic area of TuMV particles with the virus cytoplasmic inclusions, especially in the *rbohD* mutant (Figure 6C,D). A strong increase was observed in Col-0 tissues between 3 and 7 dpi, but not so strong as in *rbohD*. Even when the virus particles were not observed, TuMV epitope localization in multivesicular bodies and vacuoles were noticed, especially in the *rbohF* mutant (Figure 7A) or Col-0 (Figure 6B). Gold particles’ deposition along virus particles and around “single membrane with tubules” structures was revealed in the vacuoles of *rbohF* mutants (Figure 7B). Just a few gold granules were presented in vacuoles and around the altered cell wall in *rbohD/F* plants (Figure 7C,D). Likewise, quantification of the virus at day 3 revealed the most intense gold deposition in *rbohD*. Additionally, *rbohD* was the genotype where the depositions increased the most between 3 and 7 dpi (Figure 8). The weakest localization of TuMV was observed in *rbohF* and especially in the double mutant *rbohD/F* (Figure 7C,D and Figure 8). In addition, in both *rbohF* and *rbohD/F* the amount of localized virus decreased between days 3 and 7. These data clearly confirmed the limitation of the TuMV infection cycle in *rbohF* and *rbohD/F* mutants.

### 2.4. Distribution of Pathogenesis-Related Protein 1 (PR1) after TuMV Inoculation

**Figure 9:**
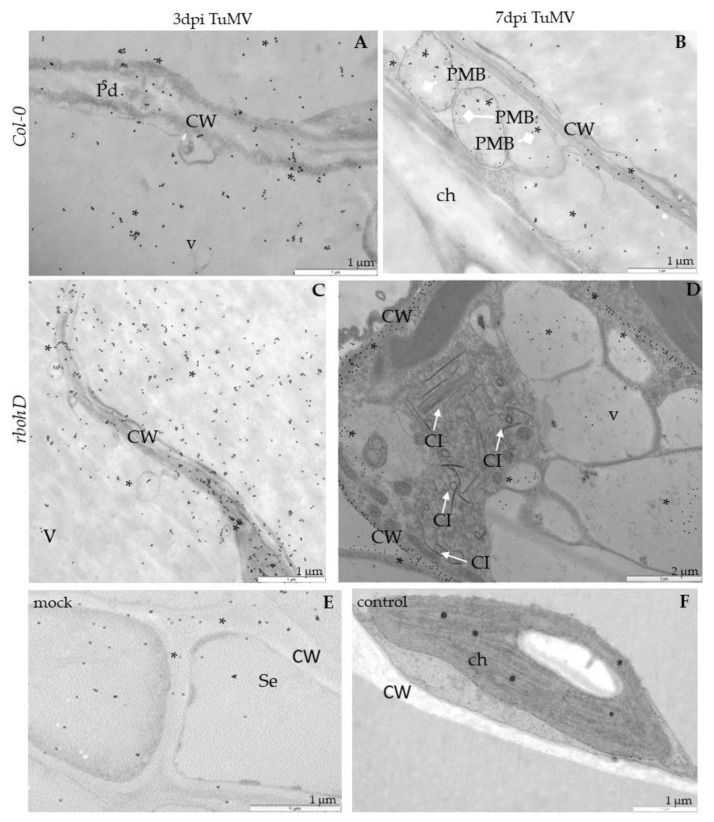
Localization of PR1 in Col-0 and *rbohD* leaf tissues 3 (**A**,**C**) and 7 days (**B**,**D**) after TuMV inoculation examined by immunogold electron microscopy. Control comprises Col-0 mock-inoculated leaf tissues (**E**) and leaf tissues without primary antibodies (**F**). (**A**) PR1 (*) in cell wall (CW), plasmodesmata (Pd) and vacuole (v) in Col-0 mesophyll 3 days after TuMV inoculation. (**B**) PR1 presence (*) in cell wall (CW), paramular bodies (PMBs; diamond head arrow) in apoplast area of palisade mesophyll Col-0 plants 7 days after TuMV inoculation. (**C**) PR1 localization (*) in cell wall (CW) and vacuoles (vs) in *rbohD* 3 days after TuMV inoculation. (**D**) PR1 deposition (*) along cell wall (CW) and in vacuoles (vs) in *rbohD* spongy mesophyll cell with virus cytoplasmic inclusions (CI with arrow). (**E**) PR1 (*) in cell wall (CW) and vacuole (v) in different type of phloem cells from mock-inoculated Col-0 plants. Se—sieve element. (**F**) Mesophyll from mock-inoculated Col-0 control plants without PR1 epitopes when primary antibodies were replaced by pre-immune serum. ch—chloroplast; CW—cell wall.

**Figure 10:**
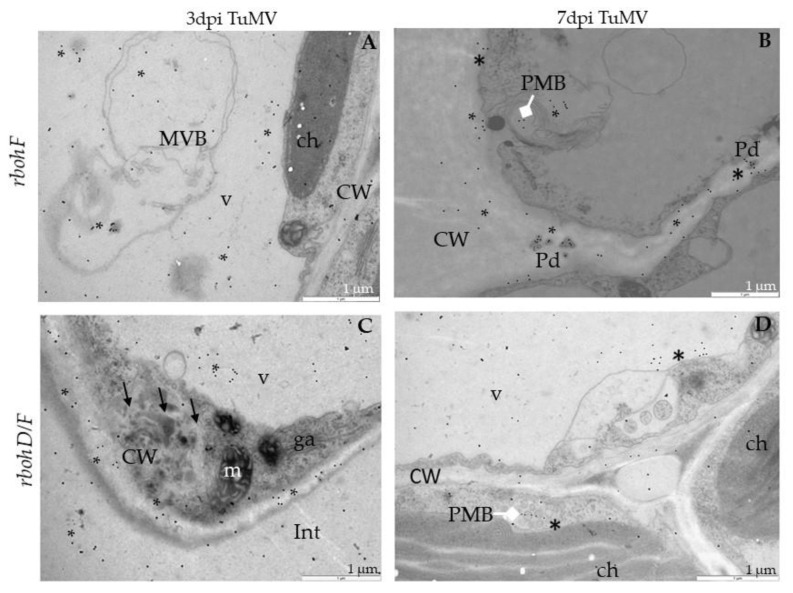
Localization of PR1 in *rbohF* and *rbohD/F* leaf tissues 3 (**A**,**C**) and 7 days (**B**,**D**) after TuMV inoculation examined by immunogold electron microscopy. (**A**) PR1 localization (*) around multivesicular bodies (MVBs) in vacuole (v) in *rbohF* palisade mesophyll cell. ch—chloroplast; CW—cell wall. (**B**) PR1 presence (*) in plasmodesmata (Pd), cell wall (CW) and paramular bodies (PMBs; white diamond head arrow) in *rbohF* phloem parenchyma cell. (**C**) PR1 (*) in and around changed (arrows) cell wall (CW) in *rbohD/F* spongy mesophyll cell. ga—Golgi apparatus, m—mitochondrion, v—vacuole. (**D**) PR1 presence (*) in paramular bodies (PMBs; diamond head arrow) and vacuole (V) in *rbohD/F* spongy mesophyll cell. ch—chloroplast; CW—cell wall; v—vacuole.

**Figure 11:**
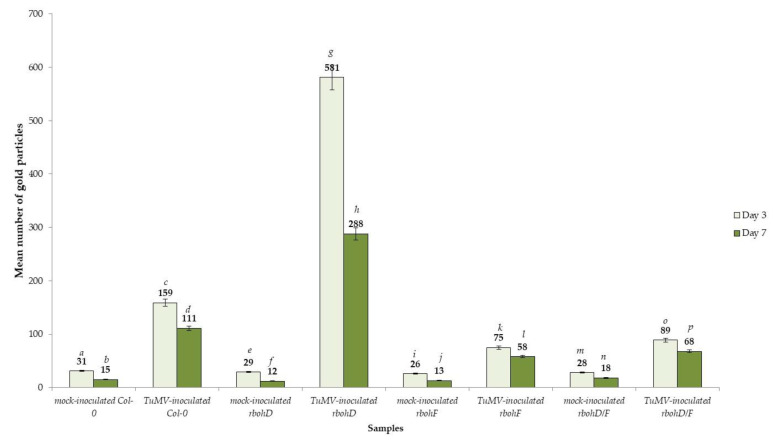
Quantification of PR1 antigen localization in mock- and TuMV-inoculated *Arabidopsis thaliana* Col-0, *rbohD*, *rbohF* and double *rbohD/F* mutant at 3 and 7 days after inoculation. Significant differences between classes at *p* < 0.05 level of significance by ANOVA with post-hoc Tukey HSD were assessed. The statistical significant values are marked by letters above chart bars.

PR1 expression is induced by TuMV infection, therefore this protein was used as a marker to indicate the progression of TuMV infection. For all four tested genotypes, TuMV inoculation increased PR1 levels (Figure 9, Figure 10 and Figure 11). Immunogold-labeled PR1 revealed the location in the cell wall and in the paramular bodies in the apoplast (Figure 9A,B) of Col-0 as an effect of TuMV inoculation. In *rbohD*, mutant PR1’s presence was noticed mainly in the cell wall, in the apoplast area and vacuoles (Figure 9C,D), whereas, in mock-inoculated leaves, weak PR1 detection in the cell wall and apoplast was found (Figure 9E), contrary to the lack of gold granules in sections where primary antibodies were replaced by pre-immune serum (Figure 9F). In *rbohF,* PR1 was primarily found in paramular bodies, but also in plasmodesmata within the cell wall (Figure 10A,B). Similarly, in *rbohD/F* the paramular bodies were decorated with gold granules, as was the reinforced cell wall in mesophyll cells (Figure 10C,D).

The green fluorescence signal localizing PR1 was mainly detected in the epidermis cell wall, but also in the trichomes cells. PR1 was also detected in vascular bundles regardless of genetic background (Figure S1 in Supplementary Materials). Analyzing the quantification of immunogold-labeled PR1, a low level of PR1 antigen in mock-inoculated Col-0 plants was revealed (Figure 11), whereas Col-0 plants displayed an over five-fold induction as compared to mock-inoculated plants at 3 dpi. Moreover, the *rbohD* mutant displayed the highest induction (20.03-fold), in contrast to PR1 *rbohF* and *rbohD/F*, which displayed the lowest induction with TuMV at 3 dpi (2.88-fold and 3.18-fold, respectively). However, the level of detectable PR1 decreased by 1.309- to 2.317-fold between 3 and 7 dpi in all mock- and virus-inoculated plants, with the lowest decrease observed in *rbohF* and *rbohD/F*. The amount of PR1 protein in the different lines correlates with the level of TuMV proliferation and the number of virus particles, suggesting that PR1 is a good marker for virus infection and that it could play a role in stopping the virus.

### 2.5. H_2_O_2_ in Response to TuMV Infection

**Figure 12:**
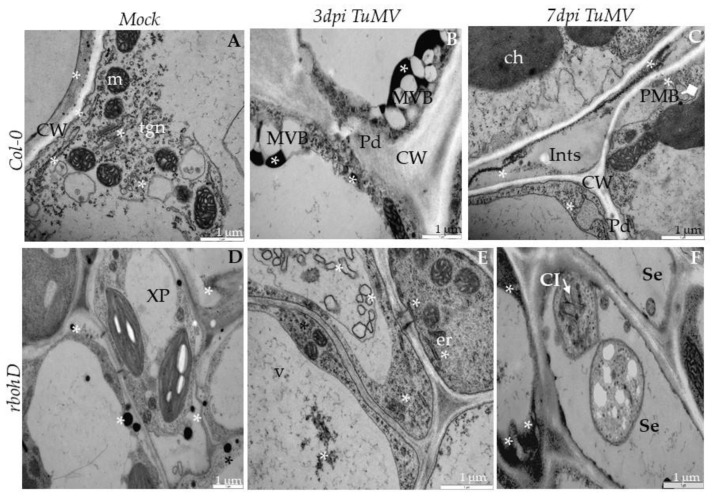
H_2_O_2_ detection in Col-0 and *rbohD* mock-inoculated leaf tissues (**A**,**D**) and 3 (**B**,**E**) and 7 days (**C**,**F**) post-TuMV inoculation examined under TEM. (**A**) Electron-dense deposits of cerium perhydroxide (*) in the cell wall and along the trans Golgi network (tgn) and vesicles in the cytoplasm of Col-0 spongy mesophyll cell. CW—cell wall; m—mitochondrion. (**B**) H_2_O_2_ (*) around multivesicular bodies (MVBs) in Col-0 epidermis. Pd—plasmodesmata; CW—cell wall. (**C**) H_2_O_2_ (*) in cell wall (CW), intercellular space (Ints) and near paramural bodies (PMB; diamond arrowhead) in Col-0 mesophyll cells. (**D**) H_2_O_2_ (*) in the apoplast between cell wall and plasmalemma in *rbohD* mock-treated xylem cells. XP—xylem parenchyma. (**E**) H2O2 (*) in vacuoles (vs), vesicular structures and endoplasmic reticulum (er) in cytoplasm in *rbohD* phloem parenchyma cells. (**F**) H_2_O_2_ (*) in phloem parenchyma cells with cytoplasmic inclusions (Cis; white arrow) of TuMV in *rbohD* sieve element (Se).

**Figure 13:**
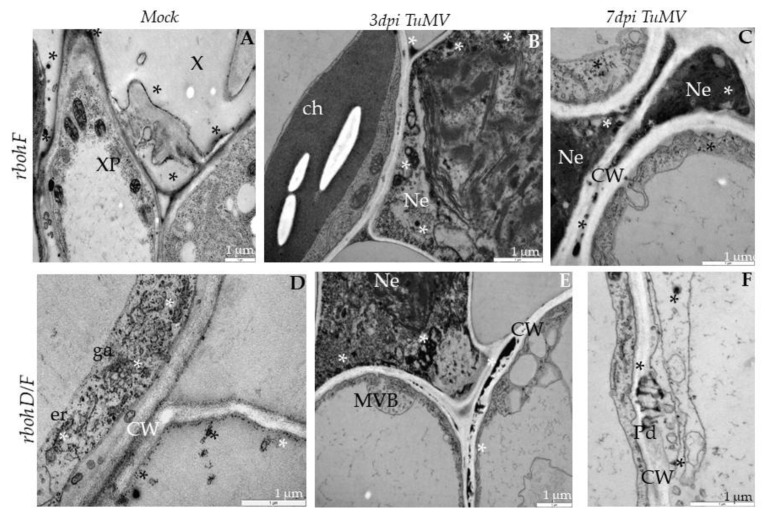
H_2_O_2_ detection in *rbohF* and *rbohD/F* mock-inoculated leaf tissues (**A**,**D**) and 3 (**B**,**E**) and 7 days (**C**,**F**) post-TuMV inoculation examined under TEM. (**A**) Cerium perhydroxide precipitates (*) in cell wall and apoplast between cell wall and plasmalemma in *rbohF* mock-inoculated xylem tracheary elements (X). XP—xylem parenchyma. (**B**) H_2_O_2_ (*) along membranous structures in necrotized (Ne) palisade mesophyll cell with destructed chloroplasts in *rbohF*. (**C**) H_2_O_2_ (*) in dark necrotized (Ne) protoplast of phloem cells and in the cytoplasm of non-necrotized phloem parenchyma cells in *rbohF*. CW—cell wall. (**D**) H_2_O_2_ staining (*) along the cell wall (CW) and endoplasmic reticulum (er) in *rbohD/F* mock-inoculated spongy mesophyll cells. ga—Golgi apparatus. (**E**) H_2_O_2_ (*) along the cell wall (CW) and membranous structures of necrotized (Ne) *rbohD/F* spongy mesophyll cell. MVBs—multivesicular bodies; CW—cell wall. (**F**) H_2_O_2_ (*) in plasmodesmata (Pd) and in cytoplasm around in *rbohD/F* mesophyll cell.

**Figure 14:**
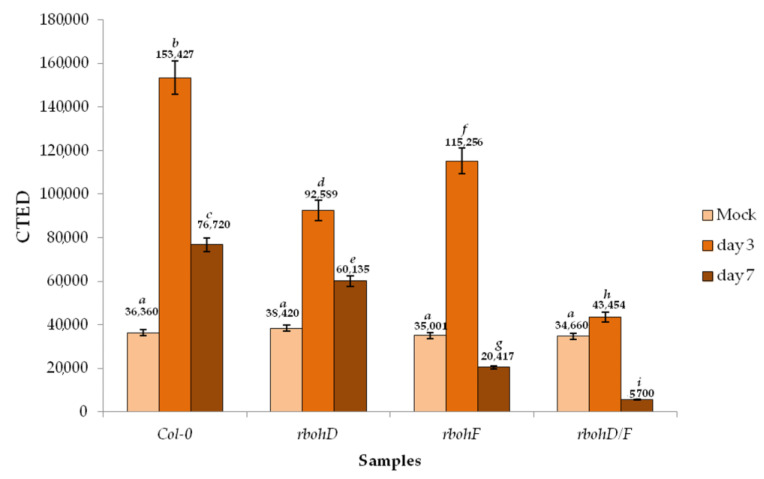
Corrected total electron density (CTED) of cerium (IV) perhydroxide precipitate in mock- and TuMV-inoculated *Arabidopsis thaliana* Col-0, *rbohD*, *rbohF* and double mutant *rbohD*/*F* at 3 and 7 dpi. Significant differences between classes at *p* < 0.05 level of significance by ANOVA with post-hoc Tukey HSD were assessed. The statistical significant values are marked by letters above chart bars.

We performed an in situ detection of H_2_O_2_ by cerium (IV) perhydroxide labeling in all four genotypes after TuMV infection and determined the corrected total electron density (CTED) based on the density of cerium perhydroxide precipitates. Analyses of the electron micrographs indicate that the H_2_O_2_ was induced by TuMV at 3 dpi as compared to mock-inoculated plants, and decreased subsequently at 7 dpi (Figure 12, Figure 13 and Figure 14). The levels of hydrogen peroxide in mock-inoculated Col-0 and in the three mutants were statistically similar. The concentration of H_2_O_2_ increased at 3 dpi, with the highest intensity in Col-0 followed by *rbohD,* and the least in *rbohD/F*. However, in Col-0 tissues the deposition of H_2_O_2_ occurred mainly along vesicular membranous structures and along the endoplasmic reticulum (Figure 12A–C). A similar pattern was observed in *rbohD* plants, although in this genotype H_2_O_2_ was also observed in vacuoles and in the apoplast (Figure 12D,F).

In contrast, in *rbohF* and *rbohD/F* H_2_O_2_ deposits were observed mainly along the cell wall (also in plasmodesmata) in the apoplast near necrotized areas (Figure 13A–F). Regardless of the genotype, H_2_O_2_ extensively decreased between 3 and 7 dpi (Figure 14), most notably in *rbohF* and *rbohD/F*. These data indicate that the two Rboh contribute to the H_2_O_2_ produced during TuMV infection, although the patterns of H_2_O_2_ driven by RbohD and RbohF seem to be different. These NADPH oxidases are responsible for most of these ROS produced after TuMV inoculation. Both RbohD and RbohF contribute to the ROS detected, but RbohD’s contribution is more important. Moreover, there are qualitative differences between the ROS that are dependent on each RBOH. Since *rbohF* and *rbohD/F* displays fewer ROS but enhanced resistance, this implies that the subset of RbohF-dependent ROS promotes susceptibility.

## 3. Discussion

### 3.1. RbohD Limits Accumulation of TuMV in Arabidopsis

Plant NADPH oxidases have emerged as important regulators of many responses to the environment, and many studies document the role of plant NADPH oxidases in different plant–pathogen interactions [36,37,38]. However, the function of these enzymes in plant–virus interactions remains poorly investigated. In this work, we focuses on two Arabidopsis NADPH oxidase genes, *RbohD* and *RbohF*, the two highest expressed homologues that are pleiotropic and act often redundantly in many immune responses to a wide range of pathogens [31,32,36]. We analyzed the responses to infection with the Turnip mosaic virus, the most threatening viral pathogen on Brassicaceae. Using Arabidopsis *rbohD* and *rbohF* transposon mutants, we demonstrated that *rbohD* supports more TuMV proliferation compared to Col-0 (and *rbohF*), especially at later times (Figure 1). Ultrastructural studies confirmed these results. TuMV infection effects were more severe in *rbohD* tissues, where TuMV induced typical potyviral cytoplasmic inclusions not only in mesophyll cells but also in phloem and xylem cells, providing evidence for systemic virus spread (Figure 3A–D). Moreover, virus particles and inclusions were observed at 3 dpi and were more intense than in the other genotypes (Figure 6A–D and Figure 7A–D). Consequently, in situ quantification of virus particles in these tissues showed that *rbohD* supports the highest accumulation of TuMV (Figure 8). Thus, these results suggest that RbohD contributes to stop TuMV proliferation and spread. Our findings are similar to those observed with other pathogens—e.g., the *rbohD* mutant was found to be more susceptible than wild type Arabidopsis to infection with different biotrophic and necrotrophic fungi and bacteria [36,37,39]. Moreover, our findings on TuMV are in accordance with the results from other potyviruses. A recent study reports that the potato RbohD orthologue limits the systemic spread of a tagged *Potato Virus Y* construct (PVY^N^-GFP) to upper (non-inoculated) leaves [40]. In view of our previous studies concerning *RbohD* in a potato–PVY^NTN^ system [41] and comparing with current data on TuMV–*rbohD* interactions, it seems clear that *RbohD* has an important contribution to the resistance response to Potyvirus. In agreement, high levels of *RbohD* transcript were associated with resistance and hypersensitive response (HR) to PVY [30]. However, *RbohD* contribution to resistance is not general to all pathosystems—e.g., *rbohD* supports less *Alternaria brassicola* biomass growth than in wild type or *rbohF,* suggesting that RbohD does not develop a specific antifungal activity and does not act as a resistance factor, but rather could play a role as a cell death regulator [42]. In other pathosystems with avirulent and virulent bacteria or with the necrotrophic fungus *Botrytis cinerea,* the responses in *rbohD* (or *rbohF*) were not accompanied by an enhanced growth of the pathogen [36,43,44]. Therefore, resistance is not a general trait associated with *RbohD*.

### 3.2. RbohF Promotes TuMV Virulence in Arabidopsis

Intriguingly, *rbohF* and *rbohD/F* display less TuMV accumulation at 3 dpi than Col-0 (Figure 1), also confirmed by the detection of TuMV in situ at the ultrastructural level (Figure 4A–D, Figure 5A–D and Figure 7A–D). After virus inoculation, *rbohF* and *rbohD/F* mutants displayed changes in their chloroplasts, induction of multivesicular structures, or the appearance of single membranes with tubular structures as an effect of TuMV inoculation (Figure 4 and Figure 5). These changes have been documented in other infections with viruses [45,46], and may be an effect of cell wall rebuilding, as we previously documented for PVY^NTN^ [47,48,49]. The cell wall rebuilding was especially noticeable in the TuMV–*rbohD/F* interaction and was often accompanied with phenolic-like compounds located inside xylem tracheary elements. Additionally, *rbohF* and especially *rbohD/F* mutants exhibited local necrosis at the 7-day time point, typical of the hypersensitive response (HR; Figure S2). Importantly, these ultrastructural changes in *rbohF* or *rbohD/F* were associated with fewer virus particles, as compared to Col-0 and *rbohD* interactions. Moreover, *rbohF* or *rbohD/F* particles were located in the vacuole and occasionally in small vacuole-like structures or vesicles in the mesophyll cell of the inoculated leaf, and no cytoplasmic inclusions were observed. This less severe TuMV infection in these mutants and the fact that virus was not able to create the inclusions needed for further spread through host cells suggests that *RbohF* promotes viral replication and increases susceptibility to TuMV. Although *RbohF* has mostly been associated with the establishment of immunity, some studies document its role in susceptibility, as *rbohF* displays increased resistance in response to several pathogens compared to the wild type [36,43]. Thus, again, resistance is not always associated with *RbohF*, and its function may vary depending on the pathosystem.

However, it is noticeable that *rbohD* and *rbohF* mutants display opposed effects in the Arabidopsis–TuMV interaction, suggesting that *RbohD* and *RbohF* play different and opposing roles in response to this virus. Usually, studies on the double mutant *rbohD/F* document an additive effect in response to pathogens as well as abiotic stress [43,50,51]. Our findings indicate that ROS produced by these two oxidases may have a qualitative (spatial or temporal) difference to signal these opposite functions.

### 3.3. RbohD and RbohF Are Responsible for Most ROS Produced during TuMV Infection

In contrast to other works, where the H_2_O_2_ level was determined macroscopically via DAB (3,3′-diaminobenzidine) application, here we quantified in situ the location of H_2_O_2_ by histochemical densitometry on the electron micrographs of infected tissues (Figure 12A–F, Figure 13A–F and Figure 14). This H_2_O_2_ accumulation after TuMV was transitory, since Col-0 showed an induction of H_2_O_2_ accumulation at 3 dpi that was reduced at 7 dpi. Production of ROS was partially compromised in the NADPH oxidase mutants, with *rbohD* showing a greater reduction in levels of H_2_O_2_ than in *rbohF*. These data suggest that RbohD is responsible for most ROS produced during TuMV infection, as has been shown in the response to many other pathogens [32,43]. This is in agreement with the higher level of expression of this homologue [29]. *rbohD/F* showed an additive reduction in ROS, suggesting than these two oxidases contribute to ROS production. A similar “gradation” in H_2_O_2_ levels in these mutants has also been documented in response to other pathogens [43,50]. Interestingly, our study revealed a different pattern of H_2_O_2_ localization between susceptible/resistant genotypes. In *Col-0* and *rbohD*, the most susceptible genotypes, H_2_O_2_ decorated the vesicular and membranous structures, and was also detected inside vacuoles and in the area between the cell wall and plasmalemma—areas where a profusion of virus particles were present (Figure 12A–F). By contrast, in *rbohF* and *rbohD/F*, the most resistant genotypes, the deposits of hydrogen peroxide were linked to the cell wall, near plasmodesmata and in the area around necrotizing cells. This pattern of H_2_O_2_ deposition evidences a possible longer distance transport of these reactive species in these genotypes [52,53] and/or a putative role in plasmodesmatal permeability, a factor that could contribute to the resistance response to the virus [54]. In addition, these resistant genotypes displayed apparent necrosis, typical of the HR-like reaction, with characteristic rebuilding of cell walls and deposition of phenolic-like compounds inside xylem elements. The commonly accepted hypothesis predicts that the suppression of virus spread and multiplication during HR is due to the necrosis, which leads to virus resistance [55]. H_2_O_2_ has been identified as a key signaling molecule promoting HR-cell death [44], with the contribution of NADPH oxidases (in *Arabidopsis* RbohD and RbohF) as the usual source of ROS defense-related ROS generation [31,36,56,57]. However, these ROS can also inhibit necrosis in some pathosystems [42,48]. Even in some plant–virus interactions, downregulation of ROS and activation of antioxidant enzymes do associate with the suppression of virus-induced necrotization and susceptibility [57,58]. In our studies, we observed that the greater reduction in H_2_O_2_ in *rbohD* correlate with the lower level of necrosis and the greatest susceptibility to TuMV, suggesting that RbohD-dependent ROS contributes to restrict TuMV infection. Similar findings, where downregulation of RBOH-dependent ROS drove the suppression of the HR, have been documented in other plant–pathogen interactions such as in response to the hemibiotrophic bacterium *Pseudomonas syringae* or the hemibiotrophic oomycete *Phytophthora infestans* [31,38]. However, in our studies, although *rbohF* also displayed lower ROS production compared to Col-0, this genotype exhibited extensive necrosis and was more resistant, which indicates that RbohF-dependent ROS contributes to negatively regulate the HR and promotes susceptibility to this virus. This reduction in Rboh-dependent ROS with an increase in the HR has also been observed in response to some pathosystems such as to an avirulent *P. syringae* strain or to the biotrophic oomycete *Hyaloperonospora arabidopsidis* [31,59]. Thus, ROS might serve different signaling functions in different types of disease resistance and in the HR. Even the dual role for the same oxidase has been suggested, with ROS produced by RbohD acting as positive or negative regulator of cell death in different cellular contexts during the same *Arabidopsis-A. brassicicola* interaction [42]. The different localization of TuMV-induced H_2_O_2_ in *rbohD* and *rbohF* mutants suggests that RbohD and RbohF drive ROS generation at different locations, and that these different pools of ROS could be responsible for these opposed functions. This may be related to their differential pattern of expression. Although both oxidases are transcriptionally upregulated by pathogens, they present a differential spatio-temporal expression patter that contributes to fine-tune Rboh-dependent ROS production [29]. Intriguingly, *rbohD/F* displayed additive reduction in H_2_O_2_ but presented the same enhanced resistance than *rbohF*. This dominant effect of the *rbohF* mutation could indicate that *RbohF* acts as a susceptibility factor. It has also been documented that *Red clover necrotic mosaic virus* (RCNMV), a Dianthusvirus, hijacks the host generation of reactive oxygen species during infections [60] while RCNMV replication proteins were associated with the ROS-generating system and triggered the ROS-burst, similarly to Brome mosaic virus replication which also depends on ROS [60]. Alternatively, since *rbohD/F* (and *rbohF*) plants displayed HR-like necrosis, the enhanced dominant resistance in *rbohD/F* could be associated with the compensatory activation of other defense pathways leading to cell death/necrosis [39].

### 3.4. PR1 Accumulation Correlates with TuMV Virulence

Pathogenesis-related proteins (PRs) are usually induced during pathogen infection and biotic stresses and are classically associated with resistance [61]. It has been long hypothesized that H_2_O_2_ is able to upregulate the defense gene PR1 [62,63]. However, our data regarding localization and quantification of *Arabidopsis* PR1 protein do not support these ideas. PR1 protein quantification indicated that PR1 deposition was induced as an effect of TuMV inoculation compared to mock-inoculated plants in all genotypes analyzed (Figure 9, Figure 10 and Figure 11). However, similarly to CTED analyses of H_2_O_2_ precipitates, PR1 depositions significantly decreased between 3 and 7 dpi. Notably, our analyses revealed that the highest buildup of PR1 was observed in the most susceptible lines, *rbohD* and Col-0, whereas the weakest accumulation of PR1 was observed in *rbohF* and *rbohD/F*, the mutants that restricted virus concentration. Thus, our studies suggest a correlation between PR1 accumulation and susceptibility. Indeed, it has been suggested in other plant–virus interactions that PR1 is rather related to susceptibility than to resistance [64]. The expression of PR1 appears to largely differ among experimental systems and seems to depend on various factors including plant species, plant organs, cell type and the tissue colonization by different pathogens. During the infection of *Capsicum chinense* Jacq. With virulent isolates of PepMMoV, the peak of PR1 expression and virus accumulation occurred at 4 dpi [65]. Noticeably, in the *Arabidopsis-A. brassicola* pathosystem, *rbohD* plants also displayed increased PR1 protein accumulation and enhanced susceptibility to this pathogen compared to Col-0 [42]. In addition, our ultrastructural studies revealed a different location of PR1 accumulation between susceptible and resistant interactions. The most susceptible interactions (Col-0-TuMV and *rbohD*-TuMV) presented strong PR1 depositions in the cell wall at the sites where virus particles and inclusions were present. However, in *atrbohF* or *atrbohD/F*, the lines where the virus infection cycle was limited, PR1 appeared around multivesicular bodies and rebuilt cell wall areas. These data suggest that more than the amount of PR1 protein itself, the areas where PR1 deposition occur are more important for the limitation of the virus infection cycle. Immunofluorescence localization of PR1 indicates the preferential accumulation of PR1 in the leaf epidermis, within the basis of trichomes, and in vascular tissues (Figure S1). This observation was similar to that of Hoegen et al. [66], who reported PR1b in epidermal cells, glandular trichomes and leaf vascular bundles. The higher accumulation of PR1 protein in the more susceptible lines was probably because of the larger amount of plant cells responding to the multiplying virulent pathogen. However, PR1 is not believed to possess antiviral activity and SA (salicylic acid)-mediated responses to incompatible virus infection are almost activated by NPR1-independent pathways [67,68]. Nevertheless, the specific localization of PR1 in the most resistant genotypes after TuMV inoculation may indicate a connection of PR1 induction with susceptibility, which may be characteristic for this pathosystem. However, further studies are needed to confirm this idea.

## 4. Materials and Methods

### 4.1. Plant Material, Virus Inoculation and DAS-ELISA Test for Virus Levels

To determine changes in viral infections induced by Turnip mosaic virus (TuMV) and related to RBOHD and RBOHF, we used *Arabidopsis thaliana* (L.) Heynh wild type (Col-0) plants and specific mutants—*Arabidopsis thaliana rbohD* (knockout mutant carrying a single *dSpm* transposon insertion in *RbohD*), *Arabidopsis thaliana rbohF* (*AtrbohF*, knockout mutant carrying a single *dSpm* transposon insertion in *RbohF*) and double *Arabidopsis thaliana rbohD rbohF* (*atrbohD/F*, double knockout obtained by crossing *atrbohF* and *atrbohD* single mutants) [43]. All homozygous mutant seeds were kindly provided from Miguel-Angel Torres laboratory. Importantly, for TuMV inoculation described below, we used 19-day-old plants without any lesions and/or alterations.

Fifty plants of Col-0 and fifty plants of each type of mutant were mechanically inoculated as described by Tomilson (1970) and Walsh and Jenner (2002) [69,70] by using the TuMV inoculum (isolate PV-0104 from Leibniz Institute, Braunschweig, Germany) in phosphate buffer [71]. Leaves of mock- and TuMV-inoculated plants were assessed for the presence of virus by using DAS-ELISA, as published before by Kozieł et al. [72], with the primary antibodies against the TuMV (Bioreba, Reinach, Switzerland), followed by purified antirabbit antibodies conjugated with alkaline phosphatase (Bioreba, Switzerland) [73]. Each repeat was performed in a new ELISA plate with samples. For each test, samples from 15 mock-inoculated wide type or mutant plants were combined separately; the same was carried out for TuMV-inoculated plants. All DAS-ELISA tests were performed using the same reagents. The readings of OD_405nm_ values were acquired after 60 min in duplicate, 3 and 7 days after inoculation. The mean OD_405nm_ values were statistically assessed with a one-factor analysis of variance (ANOVA), as described in Kozieł et al. [72] with the Statistica software (version 13.0; StatSoft and TIBCO Software Inc., Palo Alto, CA, USA). For a more precise assessment, the corrected mean OD_405nm_ values were computed as presented in [72] and used to compare the relative level of virus presence/concentration in plants. The cut-off point was also calculated by using a formula suggested by Bioreba (Switzerland) [74]:

Cut-off = mean value (of all negative-healthy plants from 3 and 7 dpi) × 3

This calculated cut-off point value was 0.1738. The readings of OD_405_ were compared to the calculated cutoff point and all OD_405_ values greater than 0.1738 were considered positive (confirmed presence of virus) [75]. Significance threshold/cut-off point values of DAS-ELISA confirmed the presence of the virus in all inoculated *Arabidopsis thaliana* plants.

### 4.2. Material Preparation for TEM Ultrastructural Analyses, Immunogold Localizations and Statistical Quantification of Both TuMV Epitopes and PR1

Approximately 15 fragments near the main vein of leaves from mock-inoculated (without symptoms, Figure S2) and TuMV-inoculated plants (with symptoms) at 3 and 7 dpi were fixed, post-fixed, dehydrated and embedded in Epon 812 (Fluka) resin according to the procedure presented in (Table S1) [47,76]. Leaf samples were collected 3 times, every time from a new group of twenty plants (in two time points, at 3 and 7 dpi) After TEM material preparation, the 50–70 nm sections from leaves were mounted on Formvar-coated copper grids (for ultrastructural analyses) or nickel grids (for immunogold analyses). The latter were treated exactly as described in [41,47]. This procedure was performed to detect the TuMV but also PR1 epitopes. Localization of TuMV and PR1 was determined separately by using different types of primary polyclonal rabbit antibodies. Firstly, the anti-TuMV antibodies were used (Bioreba, Switzerland) in a 1:50 dilution for detecting the virus, followed by the anti-PR1 antibodies (Agrisera, Vänäs, Sweden), also in a 1:50 dilution. To visualize the location of TuMV and PR1, we used a secondary antirabbit antibody conjugated with nanogold particles with sizes of 18 nm (Jackson Immuno Research Europe Ltd., Cambridgeshire, UK) in a dilution 1:100. The labeling specificity was checked by incubating grids with material from mock-inoculated plants and by omission of the primary antibody from the incubating solution. The grids were then counterstained with 1% uranyl acetate for 5 min and washed 5 times for 2 min each with distilled water. The immunogold-labeled sections on grids were examined using transmission electron microscopy (TEM, FEI M268D “Morgagni” transmission electron microscope) [47,48]. After examination of TuMV and PR1 localization, the labeling quantification of general specific localization was based on Otulak-Kozieł et al. [47]. The analysis of variance was applied to the data for the gold particle concentrations by using (ANOVA) and the post-hoc Tukey HSD test in STATISTICA software (StatSoft and TIBCO Software Inc., Palo Alto, CA, USA, version 13.0). ANOVA was used as an efficient estimator of gold labeling. For the statistical estimation of immunogold labeling, we compared infected and healthy (mock-inoculated) plants of mutants. Gold particles in cell compartments were counted in forty 10-μm images with 2 fields per image. In each combination the gold particles from 200 photos were counted for TuMV and PR1.

### 4.3. Immunofluorescence Localization of PR1

The virus- and mock-inoculated leave tissues were treated as previously described by Kozieł et al. [77]. To assess the distribution of PR1, it was localized by an immunofluorescence method according to Kozieł et al. [78]. The primary antibody targeted PR1 with the procedure described above in Section 4.2. The secondary antibody was antirabbit anti-IgG carrying the attached AlexaFluor^®^488 (Abcam, Cambridge, UK). Slides were imaged under a PROVIS AX70 fluorescent microscope with an Olympus UP90 high-definition camera (Olympus, Warsaw, Poland) and the photos were analyzed using Olympus Cell Sense Standard Software (version 1.18; Olympus, Center Valley, PA, USA).

### 4.4. Localization and Quantification of H_2_O_2_ by CTED Method

Detection of H_2_O_2_ was accomplished using CeCl_3_ as described by Bestwick et al. [79] with changes published by Otulak and Garbaczewska [80] and Kozieł et al. [72]. The leaf tissues were shortly pre-incubated in 50 mM MOPS buffer (pH 7.2) containing 5 mM CeCl_3_, washed for one hour with the same buffer, and fixed according to the procedure of Otulak and Garbaczewska [80]. Samples were contrasted and fixed in 2% (*w*/*v*) OsO_4_ in a cacodylate buffer and dehydrated in a series of ethanol–water washings, as described in Kozieł et al. [72]. The material was gradually saturated with Epon 812 (Fluka) and polymerized according to the procedure in [80]. Observations were made as previously described in [77]. To quantify the level of H_2_O_2_, a CTED method was used as presented by Kozieł et al. [72] which enables the analysis of distribution of electron-dense cerium (IV) perhydroxide precipitates. The electron density of these precipitates is directly proportional to the amount of H_2_O_2_. Then, CTED values were analyzed for statistical significance according to methodology presented by Kozieł et al. [72] with ANOVA and Tukey’s post-hoc HSD test performed using Statistica software (version 13.0; StatSoft and TIBCO Software Inc., Palo Alto, CA, USA).

## 5. Summary

In this study we compared the ultrastructural effects of TuMV infection on *A.thaliana* Col-0 and NADPH oxidase *rbohD* and *rbohF* transposon mutants and performed in situ localization and quantification of virus particles, H_2_O_2_ and PR1 proteins. TuMV infection in *rbohD* led to a systemic development of virus infection. The virus concentration and localization significantly increased between 3 and 7 days after inoculation in *rbohD*, even more dynamically than in Col-0. In this mutant, in situ localization of H_2_O_2_ correspond with virus particles and was observed in vacuoles and between the cell wall and plasmalemma. Moreover, quantification of H_2_O_2_ showed that *rbohD*-inoculated plants displayed a considerable reduction in ROS accumulation compared to Col-0. ROS derived from RbohD plays a role in the resistance response to TuMV in this pathosystem. Opposite to *rbohD*, a significant decrease in virus infection cycle was observed in *rbohF* and *rbohD/F* compared to Col-0 or *rbohD.* Interestingly, *rbohD/F* response was accompanied by local necrosis, typical for HR-like reactions, with the characteristic rebuilding of cell walls, phenolic-like compounds deposition inside xylem elements, together with the rare occurrence of virus particles and complete lack of virus inclusions. Noticeably, in situ localization of H_2_O_2_ in these more susceptible genotypes revealed that the deposits of hydrogen peroxide were differentially linked to the cell wall, near plasmodesmata and in the area around necrotizing cells. This pattern of H_2_O_2_ deposition evidenced a possible longer distance transport of these reactive species in these genotypes that could contribute to the enhanced resistance observed. Deposition of PR1 protein was activated in all *A. thaliana* variants compared to mock-inoculated plants, but the more susceptible *rbohD* plants displayed a higher level of PR1, whereas *rbohF* and *rbohD/F* displayed a lower accumulation. Our qualitative and quantitative analyses indicated that PR1 location strictly parallels the virus alterations, although it is not clear that the PR1 induction acts as an antiviral factor. The important role of ROS produced by NADPH oxidases in regulating the *Arabidopsis* response to TuMV and a differential role of RbohD and RbohF in this interaction were revealed.

Further molecular and cellular studies are needed to elucidate the possible role of other signaling molecules (e.g., nitric oxide metabolism and calcium signature distribution) that may potentially serve as active components in the *A. thaliana*–TuMV pathosystem. It has been reported that nitric oxide signaling and calcium protein kinases (among other kinases) can modulate NADPH oxidase activity; these analyses may help provide a better understanding of the mechanisms modulating *A. thaliana*–TuMV interactions.

## Figures and Tables

**Table 1:** Turnip mosaic virus (TuMV) detection assessment using double antibody sandwich enzyme-linked immunosorbent assay (DAS-ELISA) on *Arabidopsis thaliana* wild type and mutant leaves at 3 and 7 dpi. Values presented are mean optical density (OD_405nm_) values. Absence of virus is marked by (−). Presence of TuMV (+) is confirmed in samples with mean OD_405nm_ above estimated cut-off point: 0.1738.

Sample	Mean OD_405nm_	Presence (+)/Absence of the Virus (−)
buffer	0.0000	−
mock-inoculated Col-0 (3 dpi)	0.0415	−
mock-inoculated *rbohD* (3 dpi)	0.0472	−
mock-inoculated *rbohF* (3 dpi)	0.0410	−
mock-inoculated mutant *rbohD/F* (3 dpi)	0.0400	−
TuMV-inoculated Col-0 (3 dpi)	0.6550	+
TuMV-inoculated *rbohD* (3 dpi)	0.6910	+
TuMV-inoculated *rbohF* (3 dpi)	0.5700	+
TuMV-inoculated *rbohD/F* (3 dpi)	0.5000	+
mock-inoculated Col-0 (7 dpi)	0.0801	−
mock-inoculated *rbohD* (7 dpi)	0.0813	−
mock-inoculated *rbohF* (7 dpi)	0.0723	−
mock-inoculated *rbohD/F* (7 dpi)	0.0600	−
TuMV-inoculated Col-0 (7 dpi)	1.379	+
TuMV-inoculated *rbohD* (7 dpi)	2.701	+
TuMV-inoculated *rbohF* (7 dpi)	0.355	+
TuMV-inoculated *rbohD/F* (7 dpi)	0.272	+

## Supplementary Materials

Supplementary Materials can be found at https://www.mdpi.com/1422-0067/21/22/8510/s1.
