# Supplementary material for: Respiratory Burst Oxidase Homologs RBOHD and RBOHF as Key Modulating Components of Response in Turnip Mosaic Virus—Arabidopsis thaliana (L.) Heyhn System
Source: Int J Mol Sci. 2020 Nov 12;21(22):8510. doi: 10.3390/ijms21228510 (PMC7696843; doi:10.3390/ijms21228510)
Supplement: Supplementary file 1 [file ijms-21-08510-s001.pdf]

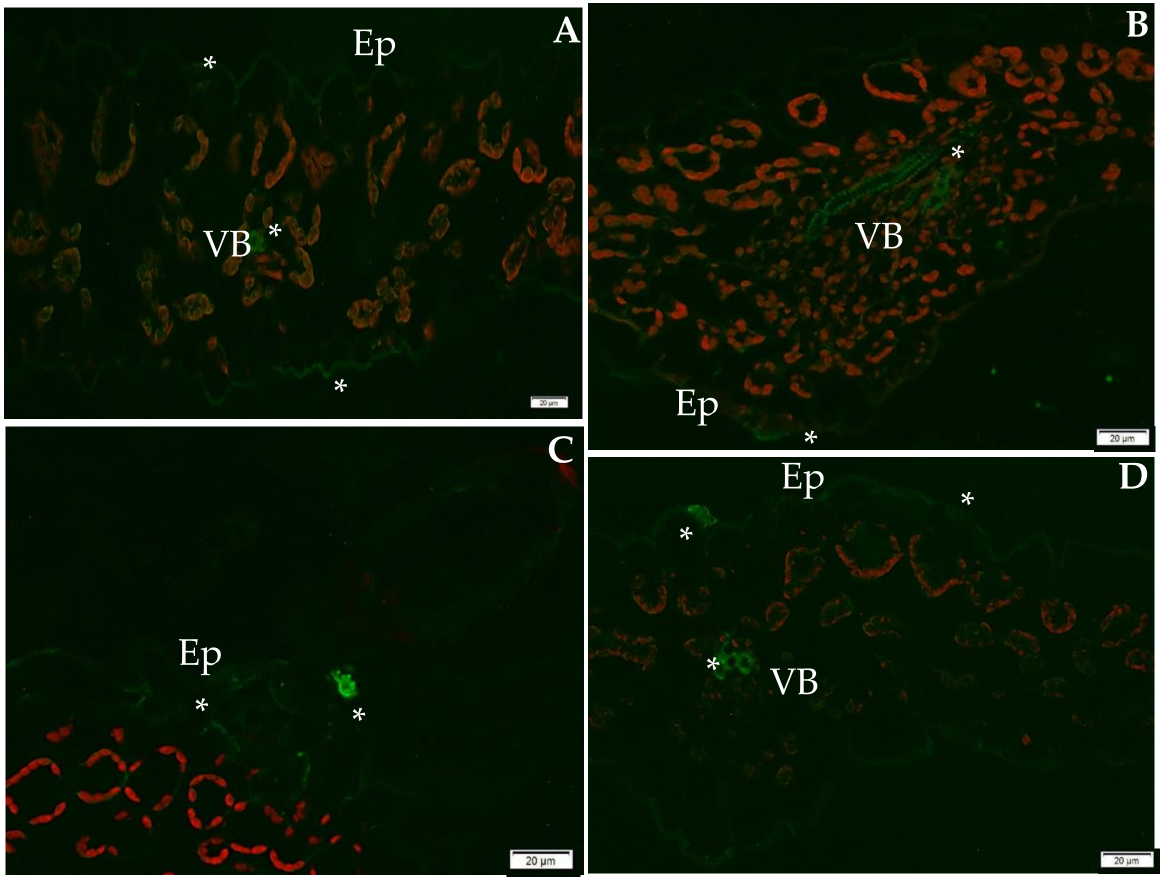


***Supplement Figure1*** **Immunofluorescence locatization of PR1 protein in Col-0 and *rbohD*, *rbohF* and *rbohD/F* leaf tissues 3 days post TuMV inoculation.** (A) Col-0 leaf, with green fluorescence signal (*) of PR1 in the epidermis and vascular bundle. (B) *rbohD, with* PR1 (*) signal in vascular bundle and lower epidermis. (C) *rbohF,* with PR1 (*) detected in the epidermis and the basis of a trichome, (D) *rbohD/F,* with PR1 in upper epidermis and vascular bundle.

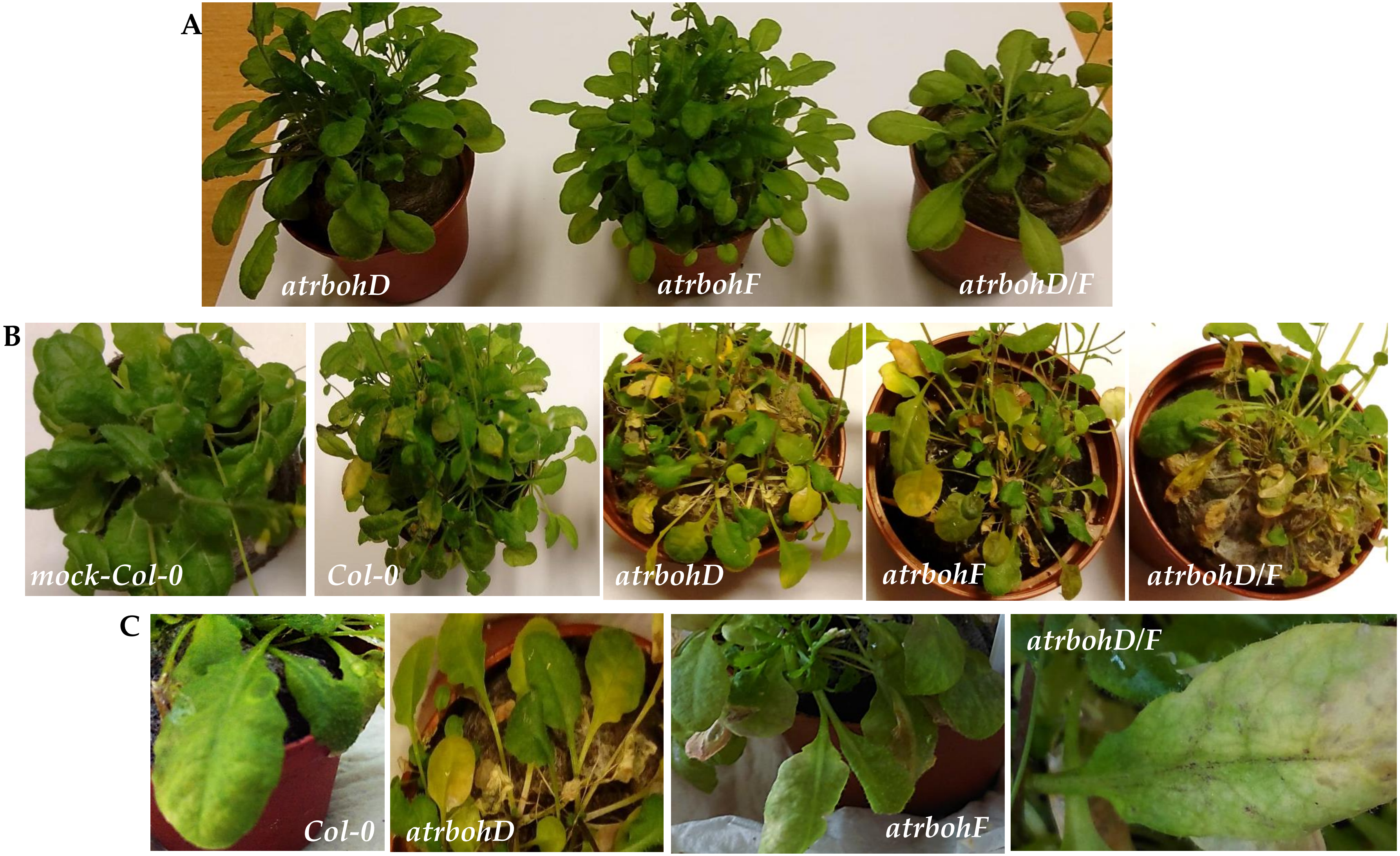


***Supplement Figure2. Symptoms of TuMV inoculation.*** ***(A)*** *Non-inoculated A.thaliana mutants.* ***(B)*** *Symptoms 7 days post mock- and TuMV- inoculation.* ***(C )*** *Leaflets 7 days post TuMV inoculation.*

**Table S1. Material preparation for transmission electron microscope (TEM) based on [36,69].**

| Step in procedure | Time |
|---|---|
| **1. Fixation** | |
| Leaf fragments were immersed in a fixative composed with 2% (w/v) paraformaldehyde and 2% (v/v) glutharaldehyde in 0.05 M sodium cacodylate buffer (pH 7.2) | 2h at room temperature |
| **2. Washing after fixation** | |
| Leaf fragments were washed 3 times in 0,05 M sodium cacodylate buffer (pH 7.2) | 3x15 min in room temperature |
| **3. Postfixation and contrasting** | |
| Leaf fragments were postfixed and contrasted in 2% (w/v) $OsO_4$ in 0.05 M cacodylate buffer (ph 7.2) | 2 h at 4°C |
| **4. Washing after postfixation** | |
| Leaf fragments were washed 3 times in 0,05 M sodium cacodylate buffer (pH 7.2) | 3x15 min at 4°C |
| **5. Dehydration** | |
| Leaf fragments were dehydrated in water-ethanol solutions with rising ethanol concentration (from 10-100%) and next in propylene oxide | dehydration in increased series of ethanol was performed 2x15 min for each ethanol solution at 4°C<br>Propylene oxide dehydration was performed 2x30 min at room temperature |
| **6. Embedding** | |
| Leaf fragments were saturated in solution of propylene oxide and Epon 812 resin in rising concentration of resin 3:1, 1:1 to 1:3 (propylene oxide : Epon 812 resin) | -For solution 3:1- 1h in room temperature in closed eppendorfs<br>-For solution 1:1-1.5h in room temperature in closed eppendorfs<br>For solution 1:3- overnight in room temperature in open Eppendorfs |
| Leaf fragments were embedded in Epon 812 resin in molds. Then plant material in molds were polymerized. | Plant's material polymerized for 24h in 60°C |
